# Association of Clinician Practice Ownership With Ability of Primary Care Practices to Improve Quality Without Increasing Burnout

**DOI:** 10.1001/jamahealthforum.2023.0299

**Published:** 2023-03-31

**Authors:** Lisa S. Rotenstein, Deborah J. Cohen, Miguel Marino, David W. Bates, Samuel T. Edwards

**Affiliations:** 1 Division of General Internal Medicine, Department of Medicine, Brigham and Women’s Hospital, Boston, Massachusetts; 2 Harvard Medical School, Boston, Massachusetts; 3 Department of Family Medicine, Oregon Health & Science University, Portland; 4 Department of Medical Informatics and Clinical Epidemiology, Oregon Health & Science University, Portland; 5 Harvard School of Public Health, Boston, Massachusetts; 6 Section of General Internal Medicine, Portland VA Medical Center, Portland, Oregon; 7 Division of General Internal Medicine and Geriatrics, Department of Medicine, Oregon Health & Science University, Portland

## Abstract

**Importance:**

Work environments and practice structural features are associated with both burnout and the ability of practices to enhance quality of care.

**Objective:**

To characterize factors associated with primary care practices successfully improving quality scores without increasing clinician and staff burnout.

**Design, Setting, and Participants:**

This cross-sectional study assessed small- to medium-sized primary care practices that participated in the EvidenceNOW: Advancing Heart Health initiative using surveys that were administered at baseline (September 2015 to April 2017) and after the intervention (January 2017 to October 2018). Data were analyzed from February 2022 to January 2023.

**Main Outcomes and Measures:**

The primary outcome of being a quality and well-being positive deviant practice was defined as a practice with a stable or improved percentage of clinicians and staff reporting burnout over the study period and with practice-level improvement in all 3 cardiovascular quality measures: aspirin prescribing, blood pressure control, and smoking cessation counseling.

**Results:**

Of 727 practices with complete burnout and aspirin prescribing, blood pressure control, and smoking cessation counseling data, 18.3% (n = 133) met the criteria to be considered quality and well-being positive deviant practices. In analyses adjusted for practice location, accountable care organization and demonstration project participation, and practice specialty composition, clinician-owned practices had greater odds of being a positive deviant practice (odds ratio, 2.02; 95% CI, 1.16-3.54) than practices owned by a hospital or health system.

**Conclusions and Relevance:**

In this cross-sectional study, clinician-owned practices were more likely to achieve improvements in cardiovascular quality outcomes without increasing staff member burnout than were practices owned by a hospital or health system. Given increasing health care consolidation, our findings suggest the value of studying cultural features of clinician-owned practices that may be associated with positive quality and experience outcomes.

## Key Points

**Question:**

What factors are associated with primary care practices successfully improving quality scores without increasing clinician and staff burnout?

**Findings:**

In this cross-sectional study of 727 small- to medium-sized primary care practices, clinician-owned practices had greater odds of improving quality without increasing burnout than practices owned by a hospital or health system.

**Meaning:**

The findings suggest the value of studying cultural features of clinician-owned practices that may be associated with positive quality and experience outcomes.

## Teaser

This cross-sectional study examines the factors associated with improving quality without increasing burnout among clinician- and non–clinician-owned primary care practices.

## Introduction

Improving the quality of preventive care and addressing burnout among health care personnel continue to be major goals for the US health care system. Currently, only 8% of US adults receive all of the high-priority, clinically appropriate preventive care services.^1^ Additionally, although more than half of US adults have risk factors for arteriosclerotic cardiovascular disease, fewer than half of these individuals achieve their clinical goals.^2^ Simultaneously, burnout has reached concerning levels among both physicians^3^ and other health care workers,^4,5^ with the COVID-19 pandemic amplifying stress and burnout across the health care workforce.^4^ Addressing burnout among health care workers is of significant interest to health care leaders, policy makers, and entities with national influence, such as the National Academies of Sciences, Engineering, and Medicine^6^ and the US Surgeon General.^7^

The association between burnout and quality of care is complex, with 1 study showing that burnout was associated with better quality^8^ and other studies suggesting no association^9,10^ or a negative association.^11,12,13,14^ For example, a 2022 study of 1064 family physicians demonstrated that patients of physicians who were experiencing burnout may have had lower ambulatory care–sensitive admissions and readmissions.^8^ On the other hand, multiple studies and a systematic review^11^ have demonstrated associations between burnout and increased perceived medical errors,^14^ worse perceptions of safety,^12^ and decreased patient satisfaction.^13^

Substantially changing quality metric performance is dependent on practice structural characteristics and the ability to change processes and capabilities as part of improvement initiatives.^15,16^ For example, regular quality improvement–focused team meetings, the presence of medical home recognition, use of standing orders, and presence of cardiovascular disease registries and electronic health record (EHR) prompts are associated with better performance on aspirin, blood pressure, and smoking cessation clinical quality measures.^17,18^ In the Comprehensive Primary Care initiative, practices adopted an average of more than 7 patient-centered medical home features; participation in the initiative was associated with improvements in chronic care and prevention scores^19^ as well as reductions in emergency department visits.^20^ In the setting of medical home transformation, practices most able to enhance diabetes outcomes had both greater structural capabilities (eg, EHRs) and distinct team, monitoring, and feedback processes.^21^

However, the practice structural features, work conditions, and initiatives that affect quality performance may also be associated with burnout among both clinicians and nonclinician practice staff.^22^ For example, prior work has demonstrated that clinician-owned or solo practices were more likely not to have burnout across clinical and nonclinical practice members.^22^ Among family physicians, insufficient time for documentation and more time spent on EHRs is associated with greater burnout.^23^ Participation in accountable care organizations (ACOs) or other demonstration projects has been associated with a higher likelihood of clinicians and practice staff experiencing burnout,^22^ and notably, some studies have suggested that primary care transformation changes associated with improved clinician burnout may contribute to the burnout of nonclinical staff.^24,25^

Given elevated burnout rates among both clinical and nonclinical health care workers^4^ and continued gaps in the quality of primary and cardiovascular preventive care,^1,2^ it is important to understand how to both enhance the quality and experience of delivering care. We assessed structural factors associated with small- and medium-sized primary care practices that took part in the EvidenceNOW: Advancing Heart Health initiative^26^ improving delivery of multiple cardiovascular quality-of-care measures without increasing burnout across their workforce.

## Methods

### Intervention, Surveys, and Sample

This cross-sectional study was a secondary analysis of data from the EvidenceNOW: Advancing Heart Health initiative.^26^ The initiative, funded by the Agency for Healthcare Research and Quality, focused on improving practice capacity and cardiovascular preventive care delivery among small- to medium-sized primary care practices between 2015 and 2019. This study was approved by the institutional review board of Oregon Health & Science University. Cooperatives administered both practice surveys and practice member surveys and obtained consent in the manner (oral or written) they determined most appropriate. This study followed the Strengthening the Reporting of Observational Studies in Epidemiology (STROBE) reporting guideline for cross-sectional studies.

The initiative involved 7 regional EvidenceNOW^26^ grantees (hereafter referred to as *cooperatives*) enrolling a total of 1513 practices across 12 states. Practices may have received several types of quality improvement support through the initiative, including practice facilitation,^23^ learning collaboratives, health information technology and support, expert consultation, and data feedback and benchmarking.^27,28^ Approaches to implementation strategies and the specific improvement resources that each practice received varied by regional cooperative.^28,29^ Clinical targets for the work were aligned with the Million Hearts initiative, which focused on metrics including (1) prescription of aspirin for patients at risk of ischemic vascular disease, (2) blood pressure control, (3) cholesterol management, and (4) provision of smoking cessation counseling (hereafter referred to as *ABCS metrics*) as a means of preventing cardiovascular morbidity and mortality.^30^ Detailed definitions of these measures are available in eTable 1 in Supplement 1, and the overall association of the intervention with quality outcomes has been previously reported.^18^

Two types of surveys were administered during the initiative. First, cooperatives administered practice surveys, which queried respondents about practice size, ownership, location, demonstration project participation, quality improvement, and EHR capabilities. Practice surveys were answered by 1 practice leader (office manager, practice owner, or lead clinician). The second type of survey was a confidential practice member survey, which was administered to all recruited practice members (both clinicians and nonclinician practice staff) within each practice. Cooperatives selected the mode in which the survey was administered (written, web-based, or telephone) to suit their practices’ needs. Both survey types were administered at baseline (September 2015 to April 2017) and after the intervention (January 2017 to October 2018).

### Measures

#### Burnout

Practice member surveys included a single-item measure of burnout, measured on a 5-category ordinal scale. This scale has been shown to correlate with the Maslach Burnout Inventory emotional exhaustion scale^31,32^ and has been leveraged in multiple large primary care studies examining workplace burnout.^10,33^ Using responses to this single-item measure, we calculated the percentage of individuals (both clinicians and staff) across each practice who met the criteria for burnout (score ≥3)^5^ at baseline and postintervention time points.^22^ We considered burnout at the practice level across job types, as a previous study demonstrated that burnout is a practice-level phenomenon associated with practice-level influences.^22^

#### Quality Measures

Quality measures were extracted from EHR-generated reports, EHR review, or health information exchange reports. Based on this information, the percentage of eligible patients in each practice that met targets related to aspirin prescription, blood pressure control, and smoking cessation counseling and/or treatment was quantified at baseline and intervention end.^34^ While practices collected and reported cholesterol management information, we did not consider the cholesterol metric in our analyses because of data quality issues and availability, as this metric was being approved by the Centers for Medicare & Medicaid Services after the start of the EvidenceNOW initiative.^26^

#### Outcome Measure

Our primary outcome for this study was a binary variable denoting practices that could be considered quality and well-being positive deviants (PDs), which we defined as those in which the proportion of individuals (both clinicians and staff) at the practice reporting burnout was stable or improved from baseline to after the intervention and that also had practice-level improvement in percentage achievement during the study period in all 3 metrics: aspirin prescribing, blood pressure control, and smoking cessation counseling. Positive deviance analysis has been used extensively in health care for the purpose of studying the relative success of specific approaches or organizations.^35^ Positive deviance approaches enable identification of characteristics or processes that facilitate the relative success identified.

#### Practice Characteristics

Practice surveys assessed practice structural characteristics (eg, size, ownership), EHR capabilities, quality reporting capabilities, participation in a demonstration project (eg, Comprehensive Primary Care initiative) at baseline, and participation in ACOs prior to the intervention’s start.^22^ Practice characteristics included cooperative practices’ affiliation (Midwest, North Carolina, Northwest, New York City, Oklahoma, Southwest, or Virginia), ownership (clinician; federally qualified health center [FQHC]; federal or Indian Health Service; hospital, health system, or health maintenance organization [HMO]; or other), location (large town, rural area, suburban, or urban core), size (1 clinician or 2-5, 6-10, or ≥11 clinicians), specialty composition (single specialty, multispecialty), and number of years under current ownership (<5, 5 to <10, 10 to <15, 15 to <20, or ≥20 years). Practice surveys additionally queried whether practices had the ability to extract EHR data, whether the vendor helped extract data and clinical quality measures, whether data on the clinical quality of care provided by the practice was publicly reported, the frequency of data discussion during practice meetings, whether the practice had EHR meaningful use certification (and if so, which stage),^36^ whether the practice was able to incorporate clinical laboratory test results as structured data, and whether the practice produced clinical quality reports on ABCS metrics in the past 6 months. For the purposes of this analysis, practice characteristics at baseline were assumed to persist at subsequent time points.

#### Practice Facilitation

Practice facilitation was 1 of the methods of support for change provided to practices in the EvidenceNOW initiative.^26^ Facilitators worked with clinicians and staff in each practice to help define and meet improvement goals^37^ and tracked the amount of facilitation they delivered to each practice.^38^ Duration of facilitator engagement and number of facilitation hours were combined into 4 facilitation dose levels, as previously described.^38^

### Statistical Analysis

Data were analyzed from February 2022 to January 2023. We first characterized practices with complete burnout and aspirin, blood pressure, and smoking quality metric information at both baseline and after the intervention. We compared the characteristics of practices with complete information with those of practices that had returned the baseline practice survey but did not have complete burnout, aspirin, blood pressure, and smoking quality metric information using χ^2^ tests for categorical variables and Kruskal-Wallis tests for continuous variables.

From among the practices with complete burnout and aspirin, blood pressure, and smoking quality metric information, we identified practices meeting the criteria to be considered a quality and well-being PD practice. We compared characteristics of these quality and well-being PD practices with those of the remaining practices with complete burnout, aspirin, blood pressure, and smoking quality metric information at both baseline and after the intervention using χ^2^ tests for categorical variables and Kruskal-Wallis tests for continuous variables.

We subsequently developed multivariable logistic regression models with SEs clustered by cooperative to identify practice-level factors associated with being a PD practice. Practice characteristics included in the multivariable model (practice location, ACO participation, demonstration project participation, and practice specialty composition) either had a theoretical basis for inclusion based on prior literature or showed distinct differences between study groups in bivariate analyses.

In sensitivity analyses, we included baseline burnout and aspirin, blood pressure, and smoking quality metric achievement in our models to assess whether the association between practice-level factors and likelihood of being a PD was affected by baseline well-being and quality performance. Finally, given expected associations of practice facilitation with both staff well-being and quality performance as well as evidence of differential engagement with practice facilitation by type of practice ownership,^39^ we conducted a sensitivity analysis to examine the extent to which adjusting for practice facilitation dose delivered affected significant associations between practice-level factors and likelihood of being a PD practice.

All analyses were conducted in SAS OnDemand for Academics (SAS Institute Inc). Statistical analyses were performed with a significance level of 2-sided *P* < .05.

## Results

Among a total of 1513 EvidenceNOW^26^ practices, we studied 1371 practices that returned baseline practice surveys (90.6% response rate). For analysis, we limited the sample to 727 practices with complete data on burnout and aspirin, blood pressure, and smoking quality metric information at baseline and postintervention (48.1% response rate based on the total sample; 53.0% response rate based on the practices that returned the baseline practice surveys). The eFigure in Supplement 1 depicts the flow of practice selection for this analysis. Among all enrolled practices, 1070 (70.7%) had both baseline and postintervention aspirin-prescribing data, 1094 (72.3%) had complete blood pressure data, and 1050 (69.4%) had complete smoking cessation counseling data.

As shown in Table 1, of the 727 practices in the analysis, 145 (19.9%) were solo practices, 356 (49.0%) had 2 to 5 clinicians, and the remaining 226 practices (31.1%) had 6 or more clinicians. A total of 311 practices (42.8%) were clinician owned; 180 (24.8%) were FQHCs; 176 (24.2%) were owned by a hospital, health system, or HMO; and 15 (2.1%) were federally owned. A majority of practices (503 [69.2%]) were located in an urban area. More than half (445 [61.2%]) of the practices were single-specialty practices, 230 (31.6%) had participated in a demonstration initiative at baseline, and 323 (44.4%) were in an ACO at baseline. Additional practice characteristics are shown in Table 1.

**Table 1.** Structural and EHR-Related Characteristics of Practices With Complete Data Considered in Analysis^a^

Characteristic	Practices, No. (%) (N = 727)^b^
**Structural characteristics**	
Cooperative	
Midwest	105 (14.4)
North Carolina	138 (19.0)
Northwest	61 (8.4)
New York City	118 (16.2)
Oklahoma	48 (6.6)
Southwest	114 (15.7)
Virginia	143 (19.7)
Practice size, No. of clinicians	
1	145 (19.9)
2-5	356 (49.0)
6-10	100 (13.8)
≥11	86 (11.8)
Missing	40 (5.5)
Practice ownership	
Clinician	311 (42.8)
FQHC	180 (24.8)
Federal, RHC, or IHS	15 (2.1)
Hospital, health system, or HMO	176 (24.2)
Missing	36 (5.0)
Other	9 (1.2)
Practice location	
Large town	83 (11.4)
Rural area	87 (12.0)
Suburban	54 (7.4)
Urban core	503 (69.2)
Specialty composition	
Single	445 (61.2)
Multiple	198 (27.2)
Missing	84 (11.6)
Time practice was under current ownership, y	
<5	114 (15.7)
5 to <10	110 (15.1)
10 to <15	99 (13.6)
15 to <20	96 (13.2)
≥20	188 (25.9)
Missing	120 (16.5)
Participation in a demonstration initiative at baseline	230 (31.6)
Practice in an accountable care organization at baseline	323 (44.4)
Facilitation dose^c^	
Low	328 (45.1)
Short	111 (15.3)
Consistent	221 (30.4)
High	27 (3.7)
Excluded	8 (1.1)
NA	32 (4.4)
**EHR characteristics**	
Ability to extract EHR data	
No	132 (18.2)
Yes	451 (62.0)
Missing	144 (19.8)
Vendor helped extract data and clinical quality measures	
Yes	323 (44.4)
No	236 (32.5)
Missing	168 (23.1)
Data on the clinical quality of care provided by the practice were publicly reported	
No	63 (8.7)
Yes	333 (45.8)
Unknown	254 (34.9)
Missing	77 (10.6)
Frequency of data discussion during practice meetings	
Often	283 (38.9)
Not regularly	223 (30.7)
Unknown	114 (15.7)
Missing	107 (14.7)
EHR meaningful use certification stage	
Neither	91 (12.5)
1	78 (10.7)
1 and 2	446 (61.4)
Missing	112 (15.4)
Practice was able to incorporate clinical laboratory test results as structured data	
No	27 (3.7)
Yes	582 (80.1)
Missing	118 (16.2)
Practice produced clinical quality reports on ABCS metrics in past 6 mo	
No	88 (12.1)
Yes	513 (70.6)
Missing	126 (17.3)
Baseline practice-level burnout, mean (SD), %	17.3 (21.5)
Baseline practice-level quality metric performance, mean (SD), %	
Aspirin prescription	62.2 (25.7)
Blood pressure control	63.5 (15.7)
Smoking cessation counseling	62.4 (32.4)

Abbreviations: ABCS, prescription of aspirin, blood pressure control, cholesterol management, and smoking cessation counseling; EHR, electronic health record; FQHC, federally qualified health center; HMO, health maintenance organization; IHS, Indian Health Service; NA, not applicable; RHC, Rural Health Care.

^a^ Complete data are defined as having complete data for burnout and aspirin, blood pressure, and smoking measures at baseline and after the intervention.

^b^ Percentages may not add to 100% because of rounding.

^c^ Low was less than 10 hours; short, 10 to less than 50 hours for less than 10 months; consistent, 10 to 50 hours for 10 or more months; high, 50 or more hours; and excluded, more than 90 hours.

Comparisons of the characteristics of the 727 practices with complete burnout, aspirin, blood pressure, and smoking data that comprised the analytic sample vs practices that completed the baseline practice survey but did not have complete outcome data (n = 644) are shown in eTable 2 in Supplement 1. Practices with complete burnout and quality outcome data had roughly similar demographic characteristics as those with incomplete data (eTable 2 in Supplement 1). Among practices with complete data compared with those with incomplete data, there was less representation of the cooperatives in Oklahoma (48 [6.6%] vs 217 [15.8%]) and the Northwest (61 [8.4%] vs 188 [13.7%]) (*P* < .001). Practices with complete data had similar mean (SD) burnout rates as those with incomplete data (17.3% [21.5%] vs 18.2% [23.1%]; *P* = .47) and similar mean (SD) aspirin-prescribing rates (62.2% [25.7%] vs 64.8% [25.2%]; *P* = .06). However, practices with complete data had slightly higher mean (SD) baseline blood pressure control rates (63.5% [15.7%] vs 61.5% [18.0%]; *P* = .003) and slightly higher mean (SD) smoking cessation counseling rates (62.4% [32.4%] vs 57.7% [31.7%]; *P* = .007). Of practices with complete burnout and aspirin, blood pressure, and smoking quality metric information at baseline and after the intervention, 18.3% (n = 133) met the criteria to be considered quality and well-being PDs.

Unadjusted differences in structural and EHR-related characteristics of quality and well-being PD practices (n = 133) vs the other practices with complete burnout, aspirin, blood pressure, and smoking data (n = 594) are shown in Table 2. We observed differences in the prevalence of quality and well-being PD practices by cooperatives; for example, PD practices compared with other practices were more likely to be part of the Midwest (25 [18.8%] vs 80 [13.5%]) and North Carolina (37 [27.8%] vs 101 [17.0%]) cooperatives. Quality and well-being PD practices additionally had significantly different ownership profiles, specifically with higher percentages of clinician ownership (74 [55.6%] vs 237 [39.9%]) and lower representation of FQHC (26 [19.5%] vs 154 [25.9%]) or hospital, health system, or HMO (25 [18.8%] vs 151 [25.4%]) ownership. Positive deviant practices additionally had significantly lower mean (SD) rates of aspirin prescription at baseline compared with other practices (55.2% [27.7%] vs 63.8% [25.0%]; *P* = .002). Baseline mean (SD) rates did not differ significantly between PD practices and other practices for blood pressure control (61.9% [14.3%] vs 63.8% [16.1%]; *P* = .15) and smoking cessation counseling (58.7% [34.5%] vs 63.2% [31.8%]; *P* = .13). Baseline mean (SD) rates of burnout across practice staff also did not differ between PD practices and other practices (18.5% [22.9%] vs 17.1% [21.3%]; *P* = .82) (Table 2).

**Table 2.** Comparison of Structural and EHR-Related Characteristics of Positive Deviant Practices vs Other Practices With Complete Data^a^

Characteristic	Practices, No. (%)^b^			*P* value^c^
	All (N = 727)	Positive deviant (n = 133)	Other (n = 594)	
**Structural characteristics**				
Cooperative				
Midwest	105 (14.4)	25 (18.8)	80 (13.5)	.03
North Carolina	138 (19.0)	37 (27.8)	101 (17.0)	
Northwest	61 (8.4)	10 (7.5)	51 (8.6)	
New York City	118 (16.2)	19 (14.3)	99 (16.7)	
Oklahoma	48 (6.6)	9 (6.8)	39 (6.6)	
Southwest	114 (15.7)	16 (12.0)	98 (16.5)	
Virginia	143 (19.7)	17 (12.8)	126 (21.2)	
Practice size, No. of clinicians				
1	145 (19.9)	30 (22.6)	115 (19.4)	.77
2-5	356 (49.0)	66 (49.6)	290 (48.8)	
6-10	100 (13.8)	16 (12.0)	84 (14.1)	
≥11	86 (11.8)	16 (12.0)	70 (11.8)	
Missing	40 (5.5)	5 (3.8)	35 (5.9)	
Practice ownership				
Clinician	311 (42.8)	74 (55.6)	237 (39.9)	.04
FQHC	180 (24.8)	26 (19.5)	154 (25.9)	
Federal, RHC, or IHS	15 (2.1)	2 (1.5)	13 (2.2)	
Hospital, health system, or HMO	176 (24.2)	25 (18.8)	151 (25.4)	
Missing	36 (5.0)	4 (3.0)	32 (5.4)	
Other	9 (1.2)	2 (1.5)	7 (1.2)	
Practice location				
Large town	83 (11.4)	18 (13.5)	65 (10.9)	.27
Rural area	87 (12.0)	18 (13.5)	69 (11.6)	
Suburban	54 (7.4)	5 (3.8)	49 (8.3)	
Urban core	503 (69.2)	92 (69.2)	411 (69.2)	
Specialty composition				
Single	445 (61.2)	93 (69.9)	352 (59.3)	.07
Multiple	198 (27.2)	28 (21.1)	170 (28.6)	
Missing	84 (11.6)	12 (9.0)	72 (12.1)	
Time practice was under current ownership, y				
<5	114 (15.7)	22 (16.5)	92 (15.5)	.96
5 to <10	110 (15.1)	20 (15.0)	90 (15.2)	
10 to <15	99 (13.6)	17 (12.8)	82 (13.8)	
15 to <20	96 (13.2)	16 (12.0)	80 (13.5)	
≥20	188 (25.9)	38 (28.6)	150 (25.3)	
Missing	120 (16.5)	20 (15.0)	100 (16.8)	
Participation in a demonstration initiative at baseline	230 (31.6)	46 (34.6)	184 (31.0)	.42
Practice in an accountable care organization at baseline	323 (44.4)	64 (48.1)	259 (43.6)	.34
Facilitation dose^d^				
Low	327 (45.0)	52 (39.1)	276 (46.5)	.45
Short	111 (15.3)	23 (17.3)	88 (14.8)	
Consistent	221 (30.4)	46 (34.6)	175 (29.5)	
High	27 (3.7)	5 (3.8)	22 (3.7)	
Excluded	8 (1.1)	0	8 (1.4)	
NA	32 (4.4)	7 (5.3)	25 (4.2)	
**EHR characteristics**				
Ability to extract EHR data				
No	132 (18.2)	28 (21.1)	104 (17.5)	.44
Yes	451 (62.0)	83 (62.4)	368 (62.0)	
Missing	144 (19.8)	22 (16.5)	122 (20.5)	
Vendor helped extract data and clinical quality measures				
Yes	323 (44.4)	66 (49.6)	257 (43.3)	.25
No	236 (32.5)	43 (32.3)	193 (32.5)	
Missing	168 (23.1)	24 (18.1)	144 (24.2)	
Data on the clinical quality of care provided by the practice were publicly reported				
No	63 (8.7)	10 (7.5)	53 (8.9)	.81
Yes	333 (45.8)	59 (44.4)	274 (46.1)	
Unknown	254 (34.9)	51 (38.4)	203 (34.2)	
Missing	77 (10.6)	13 (9.8)	64 (10.8)	
Frequency of data discussion during practice meetings				
Often	283 (38.9)	51 (38.4)	232 (39.1)	.96
Not regularly	223 (30.7)	42 (31.6)	181 (30.5)	
Unknown	114 (15.7)	22 (16.5)	92 (15.5)	
Missing	107 (14.7)	18 (13.5)	89 (15.0)	
EHR meaningful use certification stage				
Neither	91 (12.5)	14 (10.5)	77 (13.0)	.09
1	78 (10.7)	9 (6.8)	69 (11.6)	
1 and 2	446 (61.4)	94 (70.7)	352 (59.3)	
Missing	112 (15.4)	16 (12.0)	96 (16.2)	
Practice was able to incorporate clinical laboratory test results as structured data				
No	27 (3.7)	2 (1.5)	25 (4.2)	.19
Yes	582 (80.1)	113 (85.0)	469 (79.0)	
Missing	118 (16.2)	18 (13.5)	100 (16.8)	
Practice produced clinical quality reports on ABCS metrics in past 6 mo				
No	88 (12.1)	15 (11.3)	73 (12.3)	.67
Yes	513 (70.6)	98 (73.7)	415 (69.9)	
Missing	126 (17.3)	20 (15.0)	106 (17.8)	
Baseline practice-level burnout, mean (SD), %	17.3 (21.5)	18.5 (22.9)	17.1 (21.3)	.82
Baseline practice-level quality metric performance, mean (SD), %				
Aspirin prescription	62.2 (25.7)	55.2 (27.7)	63.8 (25.0)	.002
Blood pressure control	63.5 (15.7)	61.9 (14.3)	63.8 (16.1)	.15
Smoking cessation counseling	62.4 (32.4)	58.7 (34.5)	63.2 (31.8)	.13

Abbreviations: ABCS, prescription of aspirin, blood pressure control, cholesterol management, and smoking cessation counseling; EHR, electronic health record; FQHC, federally qualified health center; HMO, health maintenance organization; IHS, Indian Health Service; NA, not applicable; RHC, Rural Health Care.

^a^ Complete data are defined as having complete data for burnout and aspirin, blood pressure, and smoking measures at baseline and after the intervention.

^b^ Percentages may not add to 100% because of rounding.

^c^ For difference between positive deviant practices and other practices.

^d^ Low was less than 10 hours; short, 10 to less than 50 hours for less than 10 months; consistent, 10 to 50 hours for 10 or more months; high, 50 or more hours; and excluded, more than 90 hours.

Changes in burnout and aspirin, blood pressure, and smoking performance differed significantly for quality and well-being PD practices vs other practices (Figure). Mean (SE) absolute changes in aspirin metric performance were 10.5 (1.2) percentage points for PD practices vs 2.3 (0.6) percentage points for others (*P* < .001); mean (SE) changes in blood pressure metric performance were 7.7 (0.8) percentage points for PD practices vs 1.1 (0.5) percentage points for others (*P* < .001); and mean (SE) changes in smoking metric performance were 11.2 (1.2) percentage points for PD practices vs 4.1 (0.8) percentage points for others (*P* < .001). The mean (SE) change in burnout across practice staff over the study period was −10.6 (1.5) percentage points for quality and well-being PD practices vs 5.4 (1.1) percentage points for other practices (*P* < .001).

**Figure.**
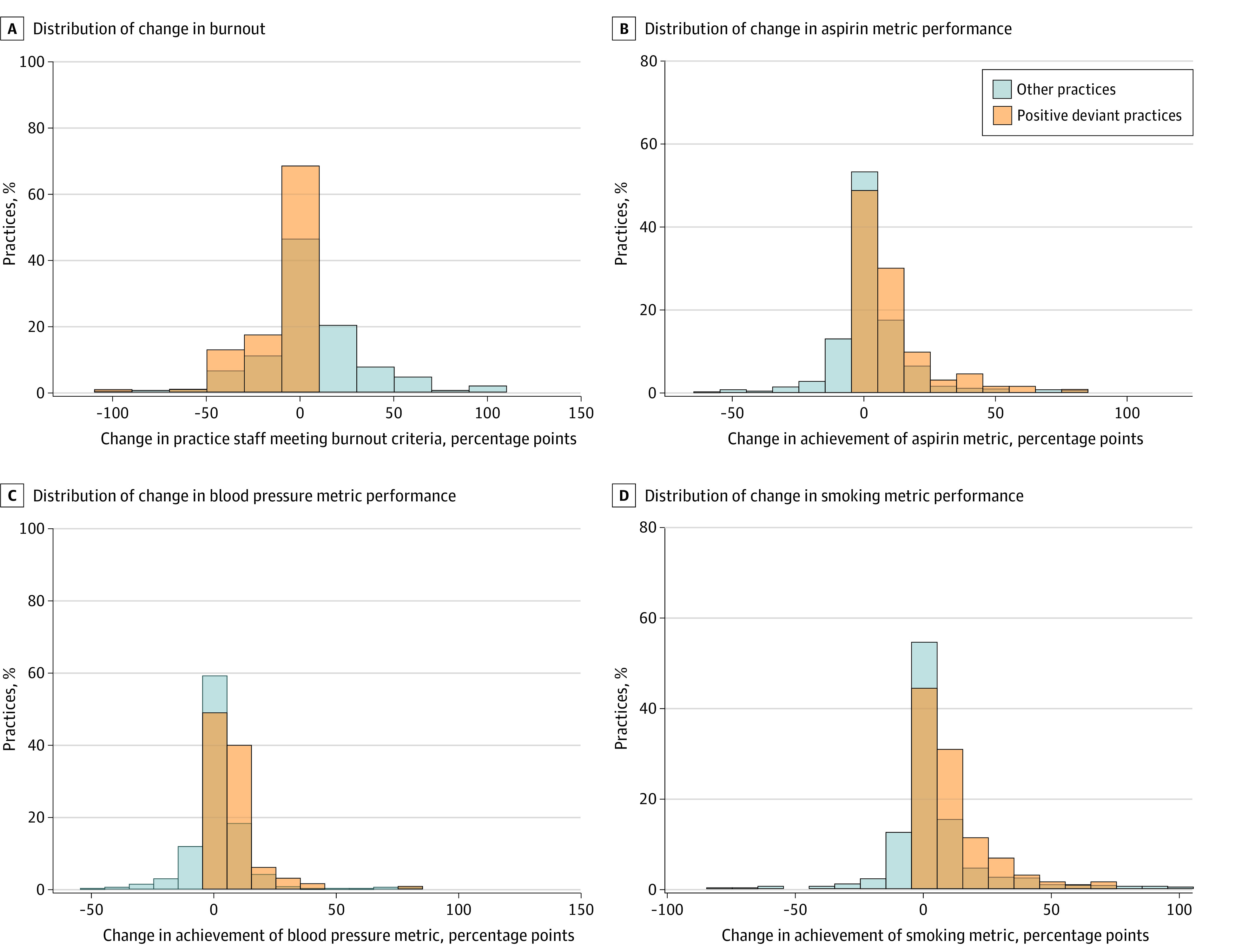
Changes in Burnout and Quality Metric Performance Over the Study Period for Positive Deviant Practices vs Other Practices

In multivariable analyses adjusting for practice location, specialty composition, participation in an ACO or a demonstration project at baseline, and cooperative, clinician-owned practices had greater odds of being able to improve quality without increasing burnout (odds ratio, 2.02; 95% CI, 1.16-3.54) than did those owned by a hospital or health system (Table 3). This translated to an adjusted estimate of 22.4% of 133 clinician-owned practices improving quality without increasing burnout compared with 12.5% of 594 practices owned by a hospital or health system. No other practice-specific factors were significantly associated with likelihood of being a quality and well-being PD practice. The significant association between clinician ownership and odds of being a PD practice persisted in sensitivity analyses adjusting for baseline practice-level burnout and aspirin, blood pressure, and smoking quality metric achievement (eTable 3 in Supplement 1), as well as in a model adjusting for facilitation dose received by the practice (eTable 4 in Supplement 1).

**Table 3.** Multivariable Logistic Regression Model Evaluating the Relative Odds of Being a Positive Deviant Practice

Characteristic	Odds ratio (95% CI)	*P* value
Practice ownership		
Clinician	2.02 (1.16-3.54)	.01
FQHC	1.15 (0.58-2.27)	.69
Federal, RHC, or IHS	1.15 (0.23-5.74)	.86
Hospital, health System, or HMO	1 [Reference]	NA
Practice location		
Large town	1.08 (0.56-2.07)	.82
Rural area	1.21 (0.63-2.29)	.57
Suburban	0.43 (0.16-1.15)	.09
Urban	1 [Reference]	NA
Practice specialty composition		
Single	1.42 (0.86-2.35)	.17
Multiple	1 [Reference]	NA
Participation in an accountable care organization at baseline	1.32 (0.86-2.02)	.21
Participation in a demonstration project at baseline	1.04 (0.68-1.59)	.86
Cooperative		
Midwest	2.63 (1.29-5.38)	.008
North Carolina	2.56 (1.28-5.13)	.008
Northwest	1.52 (0.57-4.01)	.40
New York City	1.12 (0.52-2.41)	.78
Oklahoma	1.70 (0.64-4.50)	.29
Southwest	1.13 (0.51-2.49)	.76
Virginia	1 [Reference]	NA

Abbreviations: FQHC, federally qualified health center; HMO, health maintenance organization; IHS, Indian Health Service; NA, not applicable; RHC, Rural Health Care.

## Discussion

In this cross-sectional study, we demonstrated that among 727 small- to medium-sized primary care practices, clinician-owned practices had greater odds of achieving improvements in cardiovascular quality outcomes while, on average, having decreased burnout or a relatively low level of burnout among both clinicians and staff. These findings add to a growing body of research suggesting positive satisfaction, engagement, and utilization outcomes among clinician-owned practices. In previous studies, clinician ownership has been associated with lower burnout,^5,22^ greater participation in practice facilitation,^39^ and better health care professional responses to change.^40^ Physicians working in clinician-owned practices compared with non–clinician-owned practices were more likely to be satisfied with their EHRs, even after controlling for concrete resources such as availability of staff support for documentation.^41^ In contrast, studies have suggested that hospital-owned practices have less clinician engagement^42^ and higher spending per patient,^43,44,45^ and physicians working in health systems or FQHCs described less sense of autonomy.^39^

Previous work has described perceptions of autonomy, pride in work, and control felt by physicians working in well-functioning, physician-owned practices.^46^ Our findings build on these perceptions by suggesting that the workplace cultural and structural factors present in clinician-owned practices that enhance well-being may also enable quality enhancements when coupled with experienced support and room for improvement in quality outcomes. These findings are particularly relevant in the context of increasing health care consolidation, with a recent American Medical Association report demonstrating that less than half of physicians worked in a private practice in 2022 and almost 40% of physicians worked for a practice owned in whole or in part by a hospital or health system.^47^ As clinician ownership decreases across the US, characterizing and seeking to adapt the cultural features of clinician ownership in nonownership settings may be important for sustaining both quality and workplace satisfaction.

It is notable that neither ACO participation nor participation in a demonstration project was significantly and independently associated with likelihood of being a practice that enhanced quality without increasing burnout. Additionally, despite evidence of differential engagement with facilitation by practice ownership type,^39^ adjustment for facilitation dose did not significantly affect the significant association between clinician ownership and practices improving quality without increasing burnout. Prior studies demonstrated that ACOs^48,49^ were associated with improved performance across multiple quality-of-care measures and practice facilitation was associated with improved chronic disease measures.^50^ However, in the present analysis, these factors were not independently associated with likelihood of performing well on both staff experience and quality of care when considering other practice structural factors. Given the lack of availability of nuanced information in our quantitative analysis about ACO or demonstration project participation or experiences with facilitation, these findings suggest the need for further exploration about how clinician ownership interfaces with change initiatives and supports to produce improvements in quality without worsening burnout.

To our knowledge, this study is unique in identifying factors associated with improving both quality and staff experience. Prior national initiatives to enhance primary care capabilities were associated with either small improvements in ambulatory quality measures, such as in the case of the Comprehensive Primary Care initiative^51^ or the patient-centered medical home demonstration project,^19^ or have not been meaningfully associated with ambulatory outcomes such as quality of care for individuals with diabetes and continuity of care.^20^ These initiatives were variably associated with reports of significant staff burnout and fatigue related to transformation^25^ or with minimal effects on physician experience.^52^ While prior work examined how the extent of patient-centered medical home implementation^53^ was associated with quality performance and levels of burnout and how specific elements of team-based care were associated with burnout levels,^54^ we specifically explored and identified practice features associated with the ability to improve multiple quality metrics without increasing burnout. Our work may pave the way for future exploration of organizations that enhance both experience and outcomes and reinforces the importance of measuring clinical quality and clinician and staff burnout together. This study additionally emphasizes the need for policies that facilitate provision of financial and technical support (such as through primary care extension programs^55^ that facilitate best-practice sharing) to independent primary care practices in a health care environment marked by increasing consolidation and acquisition of physician-owned practices by hospitals, large health systems, or other ownership types (eg, private equity).

### Strengths and Limitations

This study has several strengths. Using data from the EvidenceNOW initiative,^26^ we were able to examine a wide variety of practices with varying size, composition, and geography. We additionally were able to consider detailed characteristics for these practices and assess both quality and well-being outcomes over time.

The present study also has several limitations. These include the observational nature of the study; an inability to examine the association of changes in burnout with changes in all ABCS metrics due to significant missing cholesterol data; the fact that some practices faced challenges using their EHRs for quality improvement purposes even when appropriate functionality was reported as existing, suggesting nuances to capabilities reported on practice surveys^36^; and the fact that our quantitative analysis precluded explicit consideration of contextual information explored in other EvidenceNOW studies^16,29^ about individual practices and the cooperatives in which they took part, which may have influenced performance. While we were able to account for multiple practice characteristics, there may be other unobserved factors (eg, external monetary investments into participating practices, type of ACO in which a practice participated, or labor market shortages) influencing the association that we identified. Notably, the EvidenceNOW^26^ intervention focused on improvement of the ABCS metrics rather than targeting burnout specifically. Finally, practices in the intervention may have had lower burnout rates than described in contemporary estimates across physician specialties, and since burnout estimates presented are at the practice level, they represent experiences of both clinicians and staff; our findings should be interpreted bearing in mind this context.

## Conclusions

In this cross-sectional study of small- to medium-sized primary care practices that participated in the EvidenceNOW initiative,^26^ we demonstrated that clinician ownership was associated with the ability to improve multiple measures of quality without increasing burnout. Future work should examine in greater detail the cultural characteristics of clinician-owned practices that explain the association that we identified. It should additionally explore how lessons about PD practices can be leveraged to enhance quality without compromising staff experiences and how quality improvement and interventions aimed at strengthening primary care might be tailored to best fit the needs of clinician- vs non–clinician-owned practices.

## Notes

Supplement 1. **eTable 1.** Definitions of Million Hearts Initiative ABCS Quality Measures **eFigure.** Depiction of Selection Flow for EvidenceNOW Practices Included in Analysis **eTable 2.** Comparison of Practices with Complete Data for Burnout, Aspirin, Blood Pressure, and Smoking Measures to Practices with Missing Data **eTable 3.** Multivariable Logistic Regression Model Evaluating the Relative Odds of Being a Positive Deviant Practice, Including Adjustment for Baseline Burnout and Quality Metric Achievement **eTable 4.** Multivariable Logistic Regression Model Evaluating the Relative Odds of Being a Positive Deviant Practice, Including Adjustment for Facilitation Dose

Supplement 2.
Data Sharing Statement
