# Supplementary material for: Association of Clinician Practice Ownership With Ability of Primary Care Practices to Improve Quality Without Increasing Burnout
Source: JAMA Health Forum. 2023 Mar 31;4(3):e230299. doi: 10.1001/jamahealthforum.2023.0299 (PMC10066456; doi:10.1001/jamahealthforum.2023.0299)
Supplement: Supplement 1. — eTable 1. Definitions of Million Hearts Initiative ABCS Quality Measures eFigure. Depiction of Selection Flow for EvidenceNOW Practices Included in Analysis eTable 2. Comparison of Practices with Complete Data for Burnout, Aspirin, Blood Pressure, and Smoking Measures to Practices with Missing Data eTable 3. Multivariable Logistic Regression Model Evaluating the Relative Odds of Being a Positive Deviant Practice, Including Adjustment for Baseline Burnout and Quality Metric Achievement eTable 4. Multivariable Logistic Regression Model Evaluating the Relative Odds of Being a Positive Deviant Practice, Including Adjustment for Facilitation Dose [file jamahealthforum-e230299-s001.pdf]

# Supplemental Online Content

Rotenstein LS, Cohen DJ, Marino M, Bates DW, Edwards ST. Association of clinician practice ownership with ability of primary care practices to improve quality without increasing burnout. *JAMA Health Forum*. 2023;4(3):e230299. doi:10.1001/jamahealthforum.2023.0299

**eTable 1.** Definitions of Million Hearts Initiative ABCS Quality Measures
**eFigure.** Depiction of Selection Flow for EvidenceNOW Practices Included in Analysis
**eTable 2.** Comparison of Practices with Complete Data* for Burnout, Aspirin, Blood Pressure, and Smoking Measures to Practices with Missing Data
**eTable 3.** Multivariable Logistic Regression Model Evaluating the Relative Odds of Being a Positive Deviant Practice, Including Adjustment for Baseline Burnout and Quality Metric Achievement
**eTable 4.** Multivariable Logistic Regression Model Evaluating the Relative Odds of Being a Positive Deviant Practice, Including Adjustment for Facilitation Dose

This supplementary material has been provided by the authors to give readers additional information about their work.

© 2023 Rotenstein LS et al. *JAMA Health Forum*.

**eTable 1.** Definitions of Million Hearts Initiative ABCS Quality Measures[29]

| | Measure Number | Definition |
|---|---|---|
| **Aspirin Prescription** | CMS 164<br>NQF 0068 | Percentage of patients 18 years of age and older who were discharged alive for acute myocardial infarction (AMI), coronary artery bypass graft (CABG) or percutaneous coronary interventions (PCI) in the 12 months prior to the measurement period, or who had an active diagnosis of ischemic vascular disease (IVD) during the measurement period, and who had documentation of use of aspirin or another antithrombotic during the measurement period. |
| **Blood Pressure Control** | CMS 165<br>NQF 0018 | Percentage of patients 18-85 years of age who had a diagnosis of hypertension and whose blood pressure was adequately controlled (<140/90mmHg) during the measurement period. |
| **Cholesterol Management** | CMS 347 | Percentage of patients considered at high risk of cardiovascular events who were prescribed or were on statin therapy during the measurement period |
| **Smoking Cessation** | CMS 138<br>NQF 0028 | Percentage of patients aged 18 years and older who were screened for tobacco use one or more times within 24 months AND who received cessation counseling intervention if identified as a tobacco user |

© 2023 Rotenstein LS et al. *JAMA Health Forum*.

**eFigure.** Depiction of Selection Flow for EvidenceNOW Practices Included in Analysis

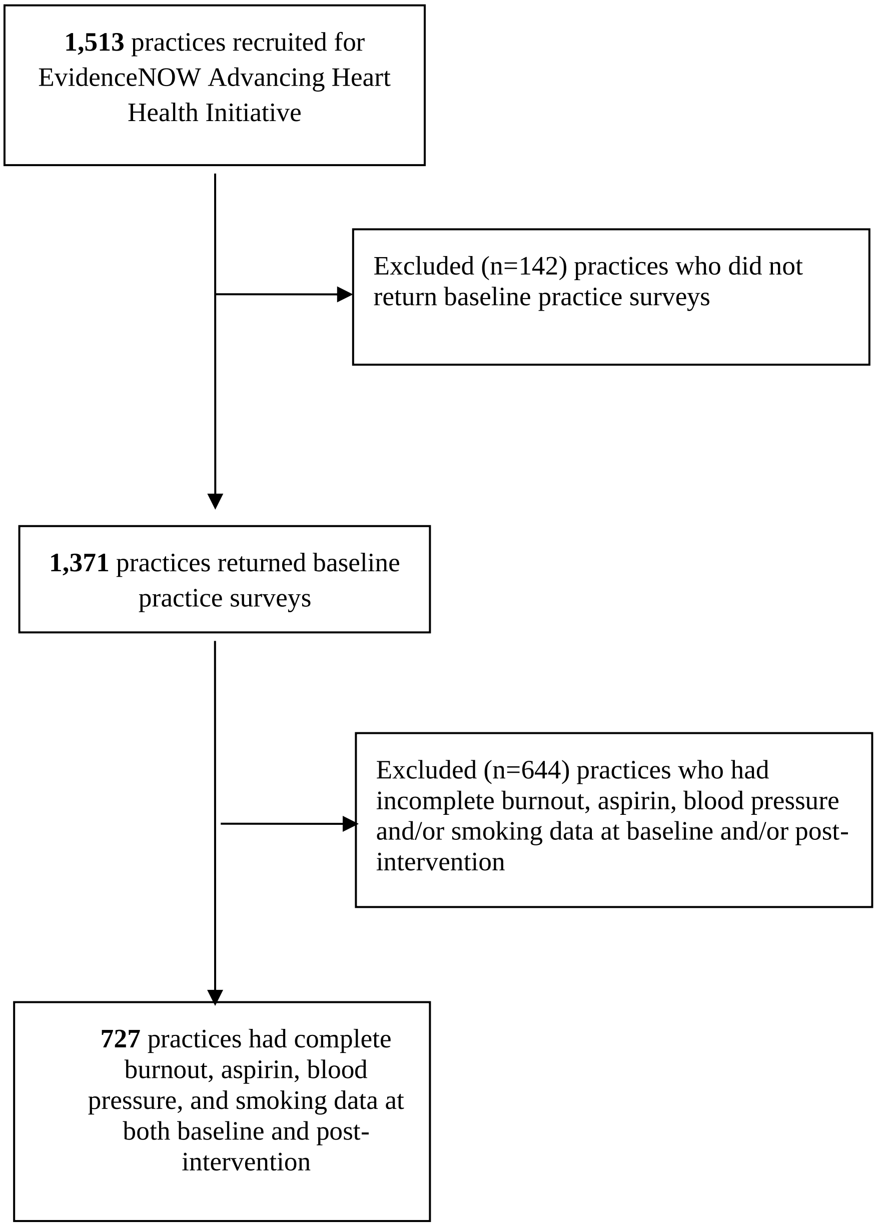


© 2023 Rotenstein LS et al. *JAMA Health Forum*.

**eTable 2.** Comparison of Practices with Complete Data* for Burnout, Aspirin, Blood Pressure, and Smoking Measures to Practices with Missing Data

| *Characteristic* | *Practices with Complete Data** <br> *(n=727)* <br> **N (%) or Mean % (SD)** | *Practices with Incomplete Data** <br> *(n=644)* <br> **N (%) or Mean % (SD)** | *p-value for Difference* |
|---|---|---|---|
| **Structural Characteristics** | | | |
| **Cooperative** | | | |
| *Midwest* | 105 (14.4) | 212 (15.5) | <0.001 |
| *North Carolina* | 138 (19.0) | 172 (12.6) | |
| *Northwest* | 61 (8.4) | 188 (13.7) | |
| *New York* | 118 (16.2) | 191 (13.9) | |
| *Oklahoma* | 48 (6.6) | 217 (15.8) | |
| *Southwest* | 114 (15.7) | 197 (14.4) | |
| *Virginia* | 143 (19.7) | 194 (14.2) | |
| **Practice Size** | | | |
| *Solo* | 145 (19.9) | 319 (23.3) | 0.03 |
| *2-5 clinicians* | 356 (49.0) | 642 (46.8) | |
| *6-10 clinicians* | 100 (13.8) | 190 (13.9) | |
| *11+ clinicians* | 86 (11.8) | 150 (10.9) | |
| *Missing* | 40 (5.5) | 70 (5.1) | |
| **Practice Ownership** | | | |
| *Clinician* | 311 (42.8) | 595 (43.4) | <0.001 |
| *FQHC* | 180 (24.8) | 268 (19.5) | |
| *Federal/RHC/IHS* | 15 (2.1) | 45 (3.3) | |
| *Hospital/Health-System/HMO* | 176 (24.2) | 363 (26.5) | |
| *Missing* | 36 (5.0) | 71 (5.2) | |
| *Other* | 9 (1.2) | 29 (2.1) | |
| **Practice Location** | | | |
| *Large Town* | 83 (11.4) | 180 (13.1) | <0.001 |
| *Rural Area* | 87 (12.0) | 211 (15.4) | |
| *Suburban* | 54 (7.4) | 98 (7.2) | |
| *Urban Core* | 503 (69.2) | 882 (64.3) | |
| **Specialty Composition** | | | |
| *Single specialty* | 445 (61.2) | 809 (59.0) | 0.06 |
| *Multi-specialty* | 198 (27.2) | 377 (27.5) | |
| *Missing* | 84 (11.6) | 185 (13.5) | |
| **Number of Years Practice Under Current Ownership** | | | |
| *<5 years* | 114 (15.7) | 223 (16.3) | <0.001 |
| *5 to <10 years* | 110 (15.1) | 222 (16.2) | |
| *10 to <15 years* | 99 (13.6) | 210 (15.3) | |
| *15 to <20 years* | 96 (13.2) | 154 (11.2) | |
| *20+ years* | 188 (25.9) | 297 (21.7) | |
| *Missing* | 120 (16.5) | 265 (19.3) | |
| **Participation in a Demonstration Initiative at Baseline** | 230 (31.6) | 182 (28.3) | 0.17 |

© 2023 Rotenstein LS et al. *JAMA Health Forum*.

| | | | |
|---|---|---|---|
| **Practice in an Accountable Care Organization at Baseline** | 323 (44.4) | 531 (38.7) | <0.001 |
| **Facilitation Dose** | | | |
| *Low: <10 hours* | 328 (45.1) | 226 (35.1) | <0.001 |
| *Short: >=10-<50 hours, <10 months* | 111 (15.3) | 116 (18.0) | |
| *Consistent: >=10-<50 hours, >=10 months* | 221 (30.4) | 226 (35.1) | |
| *High: >=50 hours* | 27 (3.7) | 51 (7.9) | |
| *Exclude, >90 hours* | 8 (1.1) | 5 (0.8) | |
| *NA* | 32 (4.4) | 20 (3.1) | |
| **EHR Characteristics** | | | |
| **Ability to Extract EHR Data** | | | |
| *No ability* | 132 (18.2) | 135 (21.0) | 0.16 |
| *Ability* | 451 (62.0) | 367 (57.0) | |
| *Missing* | 144 (19.8) | 142 (22.1) | |
| **Vendor Helps Extract Data and Clinical Quality Measures** | | | |
| *Yes* | 323 (44.4) | 262 (40.7) | 0.01 |
| *No* | 236 (32.5) | 187 (29.0) | |
| *Missing* | 168 (23.1) | 195 (30.3) | |
| **Data on the Clinical Quality of Care Provided by the Practice Publicly Reported** | | | |
| *No* | 63 (8.7) | 94 (14.6) | <0.001 |
| *Yes* | 333 (45.8) | 246 (38.2) | |
| *Don't Know* | 254 (34.9) | 229 (35.6) | |
| *Missing* | 77 (10.6) | 75 (11.7) | |
| **Frequency of Data Discussion During Practice Meetings** | | | |
| *Often* | 283 (38.9) | 189 (29.4) | 0.001 |
| *Not Regularly* | 223 (30.7) | 214 (33.2) | |
| *Don't Know* | 114 (15.7) | 137 (21.3) | |
| *Missing* | 107 (14.7) | 104 (16.2) | |
| **EHR Meaningful Use Certification** | | | |
| *Neither* | 91 (12.5) | 115 (17.9) | 0.01 |
| *Stage 1* | 78 (10.7) | 81 (12.6) | |
| *Stage 1 + 2* | 446 (61.4) | 371 (57.6) | |
| *Missing* | 112 (15.4) | 77 (12.0) | |
| **Practice Able to Incorporate Clinical Lab Test Results as Structured Data** | | | |
| *No* | 27 (3.7) | 44 (6.8) | 0.03 |
| *Yes* | 582 (80.1) | 506 (78.6) | |
| *Missing* | 118 (16.2) | 94 (14.6) | |
| **Practice Produced Clinical Quality Reports on ABCS Measures in Last 6 Months** | | | <0.001 |

© 2023 Rotenstein LS et al. *JAMA Health Forum*.

| | | | |
|---|---|---|---|
| *No* | 88 (12.1) | 142 (22.1) | 0.45 |
| *Yes* | 513 (70.6) | 323 (50.2) | |
| *Missing* | 126 (17.3) | 179 (27.8) | |
| **Baseline Burnout** | | | |
| *Practice-Level Mean % Burnout* | 17.3 (21.5) | 18.2 (23.1)<br>*n = 549* | 0.47 |
| **Baseline Quality Metric Performance** | | | |
| *Practice-Level Mean % Aspirin Prescription* | 62.2 (25.7) | 64.8 (25.2)<br>*n = 215* | 0.06 |
| *Practice-Level Mean % Blood Pressure Control* | 63.5 (15.7) | 61.5 (18.0)<br>*n = 240* | 0.03 |
| *Practice-Level Mean % Smoking Cessation Counseling* | 62.4 (32.4) | 57.7 (31.7)<br>*n* = 195 | 0.007 |

*Complete data is defined as having complete data for burnout and aspirin, blood pressure, and smoking measures at baseline and post-intervention.

© 2023 Rotenstein LS et al. *JAMA Health Forum*.

**eTable 3.** Multivariable Logistic Regression Model Evaluating the Relative Odds of Being a Positive Deviant Practice, Including Adjustment for Baseline Burnout and Quality Metric Achievement

| Characteristic | OR (95% CI) for Positive Deviance | p-value |
|---|---|---|
| **Practice Ownership** | | |
| *Clinician* | 2.16 (1.23, 3.80) | 0.008 |
| *FQHC* | 1.15 (0.58, 2.28) | 0.69 |
| *Federal/RHC/IHS* | 1.21 (0.24, 6.06) | 0.82 |
| *Hospital/Health System/HMO* | Reference | Reference |
| **Practice Location** | | |
| *Large Town* | 1.08 (0.56, 2.09) | 0.82 |
| *Rural Area* | 1.19 (0.62, 2.30) | 0.60 |
| *Suburban* | 0.41 (0.15, 1.11) | 0.08 |
| *Urban* | Reference | Reference |
| **Practice Specialty Composition** | | |
| *Single specialty* | 1.39 (0.83, 2.31) | 0.21 |
| *Multi-specialty* | Reference | Reference |
| **Participation in an Accountable Care Organization at Baseline** | 1.27 (0.82, 1.96) | 0.29 |
| **Participation in a Demonstration Project at Baseline** | 1.05 (0.68, 1.60) | 0.84 |
| **Proportion of Practice Members Reporting Burnout at Baseline*** | 1.58 (0.65, 3.88) | 0.32 |
| **Practice-Level Proportion Meeting Aspirin Quality Metric at Baseline*** | 1.01 (0.83, 1.23) | 0.91 |
| **Practice-Level Proportion Meeting Blood Pressure Quality Metric at Baseline*** | 0.43 (0.13, 1.49) | 0.19 |
| **Practice-Level Proportion Meeting Smoking Quality Metric at Baseline*** | 1.10 (0.98, 1.24) | 0.12 |
| **Cooperative** | | |
| Midwest | 2.62 (1.27, 5.40) | 0.009 |
| North Carolina | 2.45 (1.21, 4.95) | 0.01 |
| Northwest | 1.46 (0.55, 3.90) | 0.45 |
| New York | 1.17 (0.54, 2.55) | 0.69 |
| Oklahoma | 1.66 (0.62, 4.44) | 0.32 |
| Southwest | 1.09 (0.49, 2.42) | 0.83 |
| Virginia | Reference | Reference |

*Proportions represented by integer with two decimal places

© 2023 Rotenstein LS et al. *JAMA Health Forum*.

**eTable 4.** Multivariable Logistic Regression Model Evaluating the Relative Odds of Being a Positive Deviant Practice, Including Adjustment for Facilitation Dose

| Characteristic | OR (95% CI) for Positive Deviance | p-value |
|---|---|---|
| **Practice Ownership** | | |
| *Clinician* | 2.10 (1.18, 3.71) | 0.01 |
| *FQHC* | 1.33 (0.65, 2.71) | 0.43 |
| *Federal/RHC/IHS* | 1.24 (0.25, 6.20) | 0.80 |
| *Hospital/Health System/HMO* | Reference | Reference |
| **Practice Location** | | |
| *Large Town* | 1.05 (0.55, 2.03) | 0.88 |
| *Rural Area* | 1.16 (0.61, 2.21) | 0.66 |
| *Suburban* | 0.45 (0.16, 1.23) | 0.12 |
| *Urban* | Reference | Reference |
| **Practice Specialty Composition** | | |
| *Single specialty* | 1.41 (0.84, 2.36) | 0.19 |
| *Multi-specialty* | Reference | Reference |
| **Participation in an Accountable Care Organization at Baseline** | 1.32 (0.86, 2.02) | 0.21 |
| **Participation in a Demonstration Project at Baseline** | 1.01 (0.66, 1.56) | 0.95 |
| **Facilitation Dose** | | 0.96 |
| *Short: >=10-<50 hours, <10 month* | 1.23 (0.67, 2.29) | 0.51 |
| *Consistent: >=10-<50 hours, >=10 months* | 1.18 (0.62, 2.27) | 0.61 |
| *High: >=50 hours* | 1.11 (0.33, 3.69) | 0.87 |
| *Low: <10 hours* | Reference | Reference |
| **Cooperative** | | |
| Midwest | 3.41 (1.52, 7.64) | 0.003 |
| North Carolina | 3.09 (1.37, 7.00) | 0.007 |
| Northwest | 1.82 (0.62, 5.33) | 0.28 |
| New York | 1.33 (0.49, 3.66) | 0.58 |
| Oklahoma | 2.07 (0.62, 6.88) | 0.24 |
| Southwest | 1.37 (0.56, 3.33) | 0.49 |
| Virginia | Reference | Reference |

© 2023 Rotenstein LS et al. *JAMA Health Forum*.
