# Supplementary material for: Association of Clinician Practice Ownership With Ability of Primary Care Practices to Improve Quality Without Increasing Burnout
Source: JAMA Health Forum. 2023 Mar 31;4(3):e230299. doi: 10.1001/jamahealthforum.2023.0299 (PMC10066456; doi:10.1001/jamahealthforum.2023.0299)
Supplement: Supplement 2. — Data Sharing Statement [file jamahealthforum-e230299-s002.pdf]

Data Sharing Statement

Rotenstein. Association of Clinician Practice Ownership With Ability of Primary Care Practices to Improve Quality Without Increasing Burnout. *JAMA Health Forum*. Published March 31, 2023. doi:10.1001/jamahealthforum.2023.0299

Data
Data available: No

Additional Information
Explanation for why data not available: This data is under the purview of the EvidenceNOW investigators.
